# Nitrogen-Based Magneto-ionic Manipulation of Exchange Bias in CoFe/MnN Heterostructures

**DOI:** 10.1021/acsnano.2c12702

**Published:** 2023-03-30

**Authors:** Christopher J. Jensen, Alberto Quintana, Patrick Quarterman, Alexander J. Grutter, Purnima P. Balakrishnan, Huairuo Zhang, Albert V. Davydov, Xixiang Zhang, Kai Liu

**Affiliations:** † Physics Department, Georgetown University, Washington, D.C. 20057, United States; ‡ NIST Center for Neutron Research, NCNR, National Institute of Standards and Technology, Gaithersburg, Maryland 20899, United States; § Theiss Research, Inc., La Jolla, California 92037, United States; ∥ NIST Materials Measurement Laboratory, National Institute of Standards and Technology, Gaithersburg, Maryland 20899, United States; ⊥ King Abdullah University of Science & Technology, Thuwal 23955-6900, Saudi Arabia

**Keywords:** nitrogen-based magneto-ionics, exchange bias, electric field control of magnetism, ionic migration, thin-film heterostructures, spintronics

## Abstract

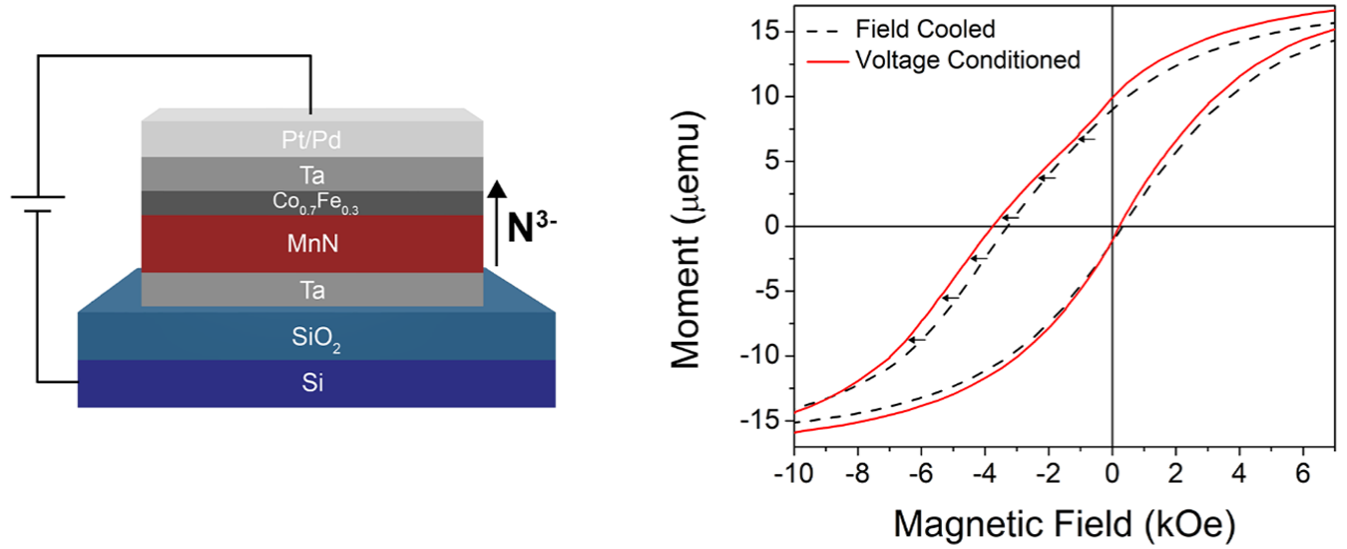

Electric field control of the exchange bias effect across ferromagnet/antiferromagnet (FM/AF) interfaces has offered exciting potentials for low-energy-dissipation spintronics. In particular, the solid-state magneto-ionic means is highly appealing as it may allow reconfigurable electronics by transforming the all-important FM/AF interfaces through ionic migration. In this work, we demonstrate an approach that combines the chemically induced magneto-ionic effect with the electric field driving of nitrogen in the Ta/Co_0.7_Fe_0.3_/MnN/Ta structure to electrically manipulate exchange bias. Upon field-cooling the heterostructure, ionic diffusion of nitrogen from MnN into the Ta layers occurs. A significant exchange bias of 618 Oe at 300 K and 1484 Oe at 10 K is observed, which can be further enhanced after a voltage conditioning by 5 and 19%, respectively. This enhancement can be reversed by voltage conditioning with an opposite polarity. Nitrogen migration within the MnN layer and into the Ta capping layer cause the enhancement in exchange bias, which is observed in polarized neutron reflectometry studies. These results demonstrate an effective nitrogen-ion based magneto-ionic manipulation of exchange bias in solid-state devices.

Electric field control of the exchange bias (EB) effect across ferromagnet/antiferromagnet (FM/AF) interfaces^1^ has offered exciting potentials for low-energy-dissipation spintronics, as it is central to spin-valve type of devices such as magnetic tunnel junctions (MTJs).^2−5^ To date, a number of approaches have shown promise in this regard, based on multiferroics,^6,7^ solid-state magneto-ionics,^8−12^ memristors,^13^ electrolytes,^14^ and spin–orbit torque.^15^ Among them, solid-state magneto-ionics is particularly appealing as it allows reconfigurable electronics by transforming the all-important FM/AF interfaces through ionic migration and enabling a wide variety of magnetic functionalities, such as magnetic anisotropy,^16−18^ antiferromagnetism,^8−10^ ferromagnetism,^19−25^ ferrimagnetic order,^11^ and Dzyaloshinskii–Moriya interaction and spin textures.^26−30^ Magneto-ionic (MI) control of EB has so far been demonstrated in several oxide systems.^8−10^ For example, in Gd/NiCoO,^9^ a FM NiCo layer was created through the spontaneous redox reaction at the interface caused by the Gd affinity to oxygen. After establishing EB between FM NiCo and AF NiCoO, oxygen ions were driven toward Gd through voltage control, resulting in an enhancement in EB.

Alternative ionic species have also been explored for magneto-ionics, such as hydrogen,^11,12,18,27,28^ nitrogen,^25,31,32^ and hydroxide,^33,34^ in the quest to overcome the limitations on room temperature ionic migration and irreversibility seen in certain oxygen-based MI systems. Initial studies in nitrogen-based magneto-ionics have demonstrated faster ionic motion,^25,31^ while also maintaining compatibility with current CMOS technology. Nitrogen diffusion and its impact on exchange bias have also been reported, where chemically induced diffusion between AF MnN and Ta seed layers have been shown to alter the resultant exchange bias in both in-plane^35−38^ and out-of-plane systems,^39^ indicating a potential magneto-ionic handle to control exchange bias.

In this work, we demonstrate a nitrogen-based magneto-ionic enhancement of exchange bias in Co_0.7_Fe_0.3_/MnN thin films that can be electrically manipulated. After films are exposed to elevated temperatures during the field cooling process, nitrogen is observed to move out of the MnN layer into both the buffer and capping Ta layers due to the Ta affinity to nitrogen. Under electric field gating, N can be driven back into the MnN layer, leading to a significant enhancement in exchange bias. This effect can be reversed under an opposite gating for a longer duration. Scanning transmission electron microscopy (STEM), X-ray diffraction (XRD), and polarized neutron reflectometry (PNR) provide direct evidence of structural and compositional changes that occur after field cooling and after voltage application. This study thus demonstrates an effective pathway for the magneto-ionic manipulation of exchange bias in solid-state configuration in a nonvolatile and energy-efficient manner.

## Results

**Figure 1:**
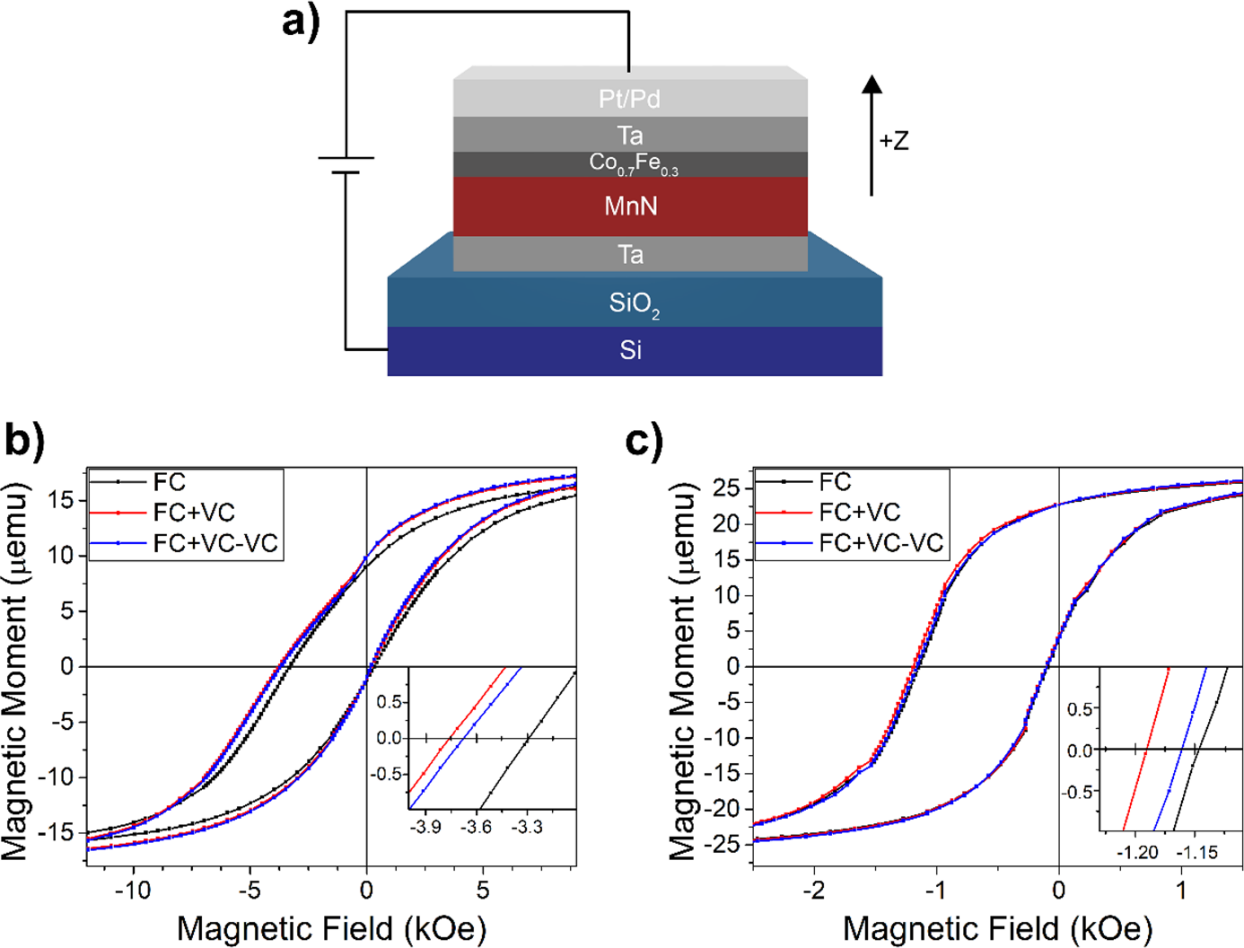
(a) Schematic diagram of the Ta/MnN/Co_0.7_Fe_0.3_/Ta/Pd(Pt) heterostructure showing positive gating convention and arrow indicating the spatial direction considered positive (+*Z*) in the heterostructure. Hysteresis loops are shown of separate Ta (10 nm)/MnN (30 nm)/Co_0.7_Fe_0.3_ (1 nm)/Ta (10 nm)/Pd (10 nm) samples at (b) 10 K and (c) 300 K. Each measurement was taken after preparing the same sample in the FC (black), FC+VC (red), and FC+VC-VC (blue) states. Insets show a zoomed-in view of the descending branch where the total magnetic moment passes through 0.

Thin film samples of Ta seed (10 nm)/MnN (30 nm)/Co_0.7_Fe_0.3_ (1 nm)/Ta (10 nm)/Pd or Pt contact (10 nm) (bottom to top structure) were grown by magnetron sputtering on thermally oxidized p-type Si substrates (SiO_2_ = 285 nm). They were field-cooled in a superconducting quantum interference device (SQUID) magnetometer from 700 K, above the Néel temperature of MnN, in a 6.5 kOe [1 Oe = 0.1 mT/μ_o_] in-plane magnetic field to 300 or 10 K. Hysteresis loops were measured for samples in the as-grown (AG), field cooled (FC), and subsequent voltage conditioned (VC) states (FC+VC and FC+VC-VC). Here, FC+VC refers to a positive gating of 20 V for 1 h after field cooling. The FC+VC-VC refers to the subsequent negative biasing for 1 h after the initial positive gating. Voltage was chosen to produce a ∼0.6 MV/cm electric field, shown to be strong enough to promote changes during sample optimization, and demonstrated to be sufficient in driving ionic migration in other magneto-ionic systems.^9,17,18^ Positive and negative bias refers to having the positive connection on the top contact and the conducting p-type Si substrate, respectively. Figure 1a illustrates the sample structure with the positive gating convention. Further details are provided in Methods.

The AF MnN chosen here is the θ-phase Mn_6_N_5+*x*_, where *x* ≥ 0, with a face-centered tetragonal (fct) structure (*F*4/*mmm*)^40^ and a Néel temperature of *T*_N_ ∼ 660 K.^41,42^ The lattice parameters of θ-MnN depend on the N concentration, with increasing N incorporation leading to larger *a* and *c* lattice constants.^40,43^ Another AF phase, η-Mn_3_N_2_, shares a similar structure with the θ-phase, except for a lack of N in one out of every three atomic planes along the *c*-axis.^40,42^ η-Mn_3_N_2_ has a *T*_N_ ∼ 913–927 K and has the same spin ordering as θ-MnN.^42^

### Magnetometry

Magnetic hysteresis loops measured at 10 and 300 K for the FC (black), FC+VC (red), and FC+VC-VC (blue) states are shown in Figure 1b,c, respectively. The FC sample exhibits a large exchange field (*H*_E_) of 1484 Oe at 10 K (Figure 1b) and 618 Oe at 300 K (Figure 1c). After positively biasing the samples (FC+VC state), *H*_E_ increases from 1484 to 1769 Oe (Δ*H*_E_ = 285 Oe) at 10 K, a 19% increase, and from 618 to 646 Oe (Δ*H*_E_ = 28 Oe) at 300 K, a 5% increase. After a subsequent reverse biasing to the FC+VC-VC state, *H*_E_ decreases in both cases (to 1741 Oe at 10 K and 631 Oe at 300 K). At 300 K, *H*_E_ of the FC state could essentially be recovered by negatively biasing for twice the duration (shown in Supporting Information Figure S1). Following this recovery, applying another positive bias leads to an increase in *H*_E_ again.

For a 1.0 nm thick Co_0.7_Fe_0.3_ film with a 0.25 cm^2^ sample area, using the saturation magnetization value of 1510 emu/cm^3^ [1 emu/cm^3^ = 1 kA·m^–1^], a saturation moment of *m*_s_ = 38 μemu is expected. In the AG state, *m*_s_ = 31 μemu, which is an ∼18% reduction from the expected magnetic moment. Since the surface roughness of MnN, probed by X-ray reflectivity, is smaller than the Co_0.7_Fe_0.3_ thickness, this reduction in *m*_s_ is not likely caused by any discontinuous FM layer. After field-cooling, a further *m*_s_ decrease to 28 μemu is observed. Both decreases in *m*_s_ are attributed to interfacial mixing at the MnN/Co_0.7_Fe_0.3_/Ta interfaces, observed in high-angle annular dark-field scanning transmission electron microscopy (HAADF-STEM) imaging, which is discussed in more detail later. However, no more substantial *m*_s_ changes are seen for the FC+VC or FC+VC-VC states, where *m*_s_ remains at 28 μemu. This fact suggests that the observed EB changes may be related to modifications in the MnN layer. To better understand the role the Ta capping layer plays in EB for this system, a reference sample of the same structure, but without the Ta capping layer, was measured in the FC state (shown in Supporting Information Figure S2). Under the same field cooling conditions, the reference sample shows almost no EB, indicating that the Ta capping layer plays a role in the initial EB, and may contribute to the enhancement seen in the FC+VC state.

### X-ray Diffraction

**Figure 2:**
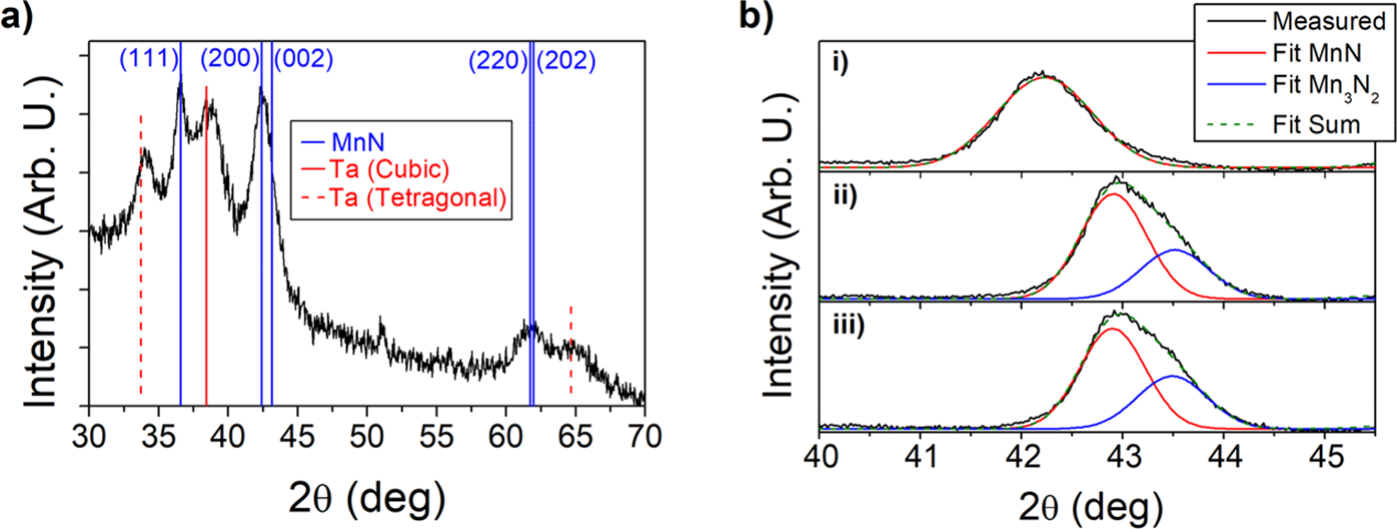
(a) Grazing incidence X-ray diffraction (GIXRD) of Ta (10 nm)/MnN (30 nm)/Ta (10 nm). Blue solid lines correspond to tabulated peaks for the θ-phase of MnN,^43^ solid red lines for the cubic phase of Ta, and dashed red lines for the tetragonal phase of Ta. (b) X-ray diffraction θ–2θ scan of Ta (10 nm)/MnN (30 nm)/CoFe (1 nm)/Pd (10 nm) in the (i) AG, (ii) FC, and (iii) FC+VC states. The black line is the measured data, the red and blue line represents the Gaussian fit for the peak associated with MnN and Mn_3_N_2_, respectively, and the green dashed line shows the sum of the two fitting curves. (i) Only fit with one Gaussian, corresponding to MnN.

XRD measurements were used to investigate the film crystallinity in the AG, FC, and FC+VC states. The lattice parameters of MnN are known to sensitively depend on the N content; e.g., the *c*-lattice constant increases with nitrogen content, with values ranging from 4.19 to 4.26 Å and beyond.^35,43^ To eliminate peak overlap between Pd(111), CoFe(200), and MnN(200) and (002), reference samples of SiO_2_/Ta (10 nm)/MnN (30 nm)/Ta (10 nm) were prepared using identical growth conditions. Grazing incidence XRD (GIXRD) confirms the θ-phase of MnN,^43^ with (111), (200)/(002), and (220)/(202) peaks at 36.5, 42.5, and 61.7°, respectively (Figure 2a). All extracted lattice parameters are in the range of 4.25–4.28 Å, with measurement uncertainty limiting the determinations of tetragonal distortions of the face-centered cubic (fcc) lattice structure seen in θ-MnN.^39,40^ The lattice parameters *c* and *a* in our system are equivalent from GIXRD, with *c*/*a* ∼ 1. Previously, *c*/*a* ratios of <1 are reported in θ-MnN systems with N atom % ≤ 50%,^39−41^ and *c*/*a* > 1 are reported as N atom % increases.^34−37^ Thus, the *c*/*a* ratio would indicate a N concentration of ≥ 50 atom % in the MnN layer of our structure. Additional contributions from Ta are also observed, including tetragonal Ta(002) and (413) at 33.98 and 64.72°, respectively (PDF 00-025-1280), body-centered cubic (bcc) Ta(110) at 38.62° (PDF 00-004-0788), and a small Ta_2_O_5_(220) peak at ∼51.0° (PDF 00-018-1304).

Additionally, θ–2θ scans were collected on a sample without the Ta capping layer, i.e., Ta (10 nm)/MnN (30 nm)/Co_0.7_Fe_0.3_ (1 nm)/Pd (10 nm), as shown in Figure 2b for the 2θ = 40.0–45.5° range (full scan is shown in Supporting Information Figure S2). This structure was chosen to avoid the observed Pd(111) peak when grown on the Ta capping layer, which overlaps with MnN(200)/(002). Instead, when grown directly on the 1 nm Co_0.7_Fe_0.3_ layer, the Pd(200) texture is promoted (PDF 00-046-1043). In the AG state (panel i), the (200)/(002) θ-MnN peak is centered around 42.22°. This peak may be indexed as MnN(200) or (002) as no appreciable difference was seen between the (200) and (002) reflections from GIXRD. This corresponds to an out-of-plane lattice constant of 4.28 Å, which places the AG MnN in the upper limit of the θ-MnN in terms of N content.^40^

For the FC sample that has been exposed to 700 K while setting the EB, the MnN(200)/(002) peak exhibits a clear shift to 43.06°, along with an asymmetric shape [panel (ii)]. This peak can be deconvoluted into two Gaussian peaks at 42.91° and 43.52°, suggesting two MnN phases. The 42.91° peak corresponds to MnN with a lower N concentration compared to the AG state.^35,39,40^ The latter peak at 43.52° is likely the (200)/(006) peak of the η tetragonal phase of Mn_3_N_2_ with nonstoichiometric N concentration, as their nominal positions are at 43.05 and 44.83°, respectively (PDF 00-001-1158). It is known that θ-MnN first decomposes into η-Mn_3_N_2_ when annealed in vacuum, leading to a decrease in N content.^43^ Also, this η-phase shares the same crystalline space group as θ-MnN (*F*4/*mmm*), where the only structural difference in the η-Mn_3_N_2_ is the lack of N in one out of every three atomic planes along the *c*-axis,^42^ leading to a unit cell that is comparable to three θ-MnN unit cells stacked along *c*. After voltage conditioning the sample, no appreciable further changes were found in the fitted peak positions or peak width (panel iii), each within the fitting error of the FC state. These findings will be further discussed in the electron microscopy and polarized neutron reflectometry sections below.

### Electron Microscopy

**Figure 3:**
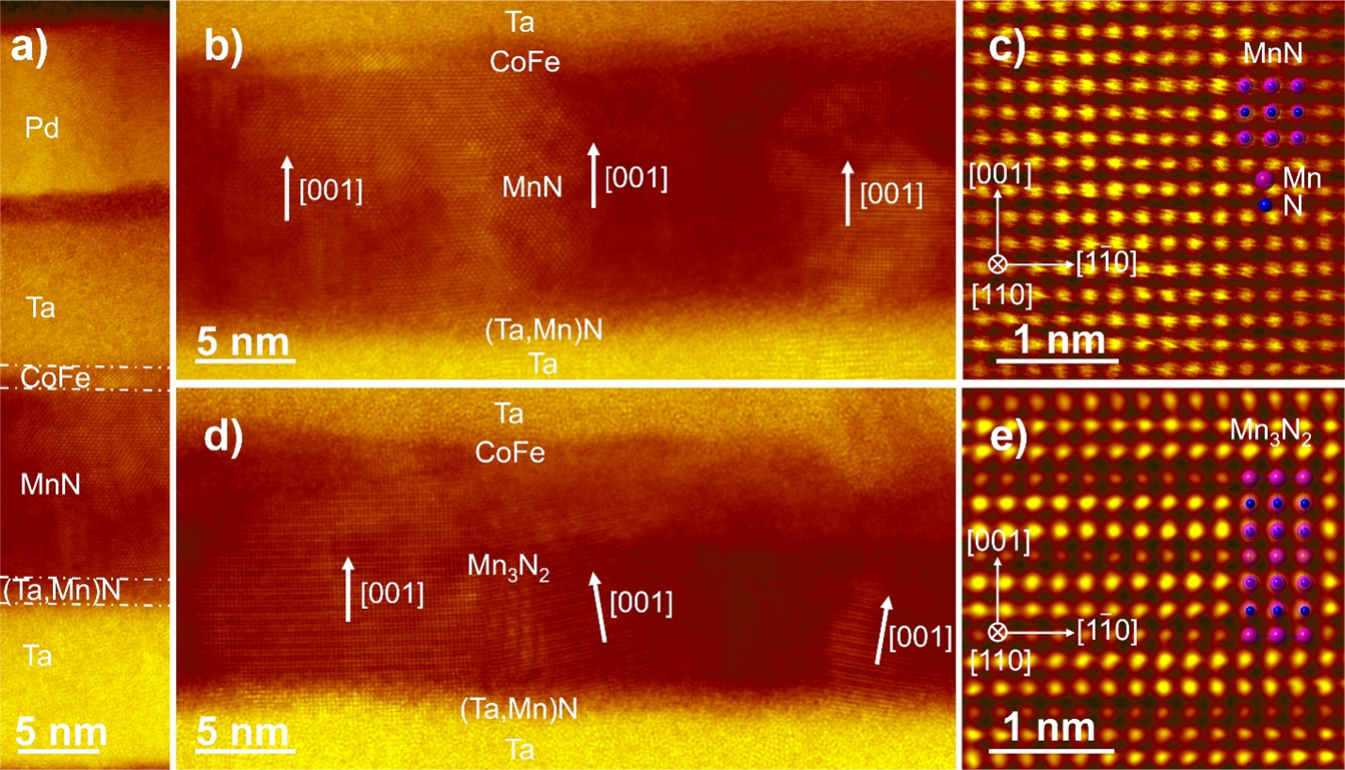
HAADF-STEM images of Ta (10 nm)/MnN (15 nm)/Co_0.7_Fe_0.3_ (1 nm)/Ta (10 nm)/Pd (10 nm) for (a) the full structure, (b,c) AG state, and (d,e) FC state. (a) Dashed lines indicate the interface between Ta/MnN at the bottom and MnN/Co_0.7_Fe_0.3_/Ta at the top. For the AG state, (b) arrows indicate the [001] texture of MnN and (c) shows the [010] zone-axis atomic structure of MnN. For the FC state, (d) arrows indicate the [001] texture of Mn_3_N_2_ and (e) shows the [010] zone-axis atomic structure of Mn_3_N_2_.

High-angle annular dark-field scanning transmission electron microscopy imaging and STEM-EELS (electron energy-loss spectroscopy) line-scan analysis were taken on Ta (10 nm)/MnN (15 nm)/Co_0.7_Fe_0.3_ (1 nm)/Ta (10 nm)/Pd (10 nm) samples in the AG, FC, and FC+VC states. Figure 3a shows a typical cross-sectional image of the AG multilayer stack. Analysis of the atomic images shows that θ-MnN is crystallized in the AG state with a [001] texture (Figure 3b,c). In addition, a (Ta,Mn)N mixture is present at the bottom Ta/MnN interface of the stack. A mixture of Fe, Co, Mn, Ta, and N in the top Ta/CoFe interface is also observed, which accounts for the *m*_s_ reduction observed from magnetometry discussed earlier. In the FC state, the MnN layer partially transformed to η-Mn_3_N_2_ which are predominantly out-of-plane ordered along [001] (Figure 3d), suggesting that the deconvoluted peak in the XRD analysis is indeed the (200)/(006) reflections of the η-Mn_3_N_2_ phase. Figure 3e displays an atomic image from a FC grain, which matches well with the overlapped atomic model of the η-Mn_3_N_2_ phase, lacking N in every third atomic layer, as compared with the θ-MnN in Figure 3c. In the FC+VC state, Mn_3_N_2_ grains remained present in the MnN layer, and no significant crystalline changes were observed with HAADF-STEM.

STEM-EELS line-scan profiles, collected across the sample thickness, helped to identify changes in N concentrations in each layer (Supporting Information Figure S3). The elemental concentrations for Co, Fe, Mn, and N in the AG state reveal a higher relative N concentration in the MnN layer compared to the Ta layers. In the FC state, this N distribution is altered, and the relative concentration in MnN decreases compared to the Ta layers. This is a manifestation of N moving out of MnN and into the Ta capping and seed layers. After gating the samples, no significant change in N concentration is observed with EELS.

These studies show that substantial changes occur in both the crystalline structure and N concentration in the MnN layer when the sample is field cooled from 700 K. In addition to the reduction of N content in MnN seen by EELS, the formation of Mn_3_N_2_ grains does confirm that the net N concentration in the MnN layer is decreasing, though the impact of these grains on EB is less clear. It is expected that Mn_3_N_2_ may not contribute strongly to the uniaxial anisotropy after field cooling despite being an AF, as field-cooling from 700 K is below its reported Néel temperature. Gating the sample did not produce changes that were observable with electron microscopy, indicating that the enhanced EB observed after gating is not due to significant structural changes in the heterostructure or from substantial N concentration changes at the level observable by EELS.

### Polarized Neutron Reflectometry

**Figure 4:**
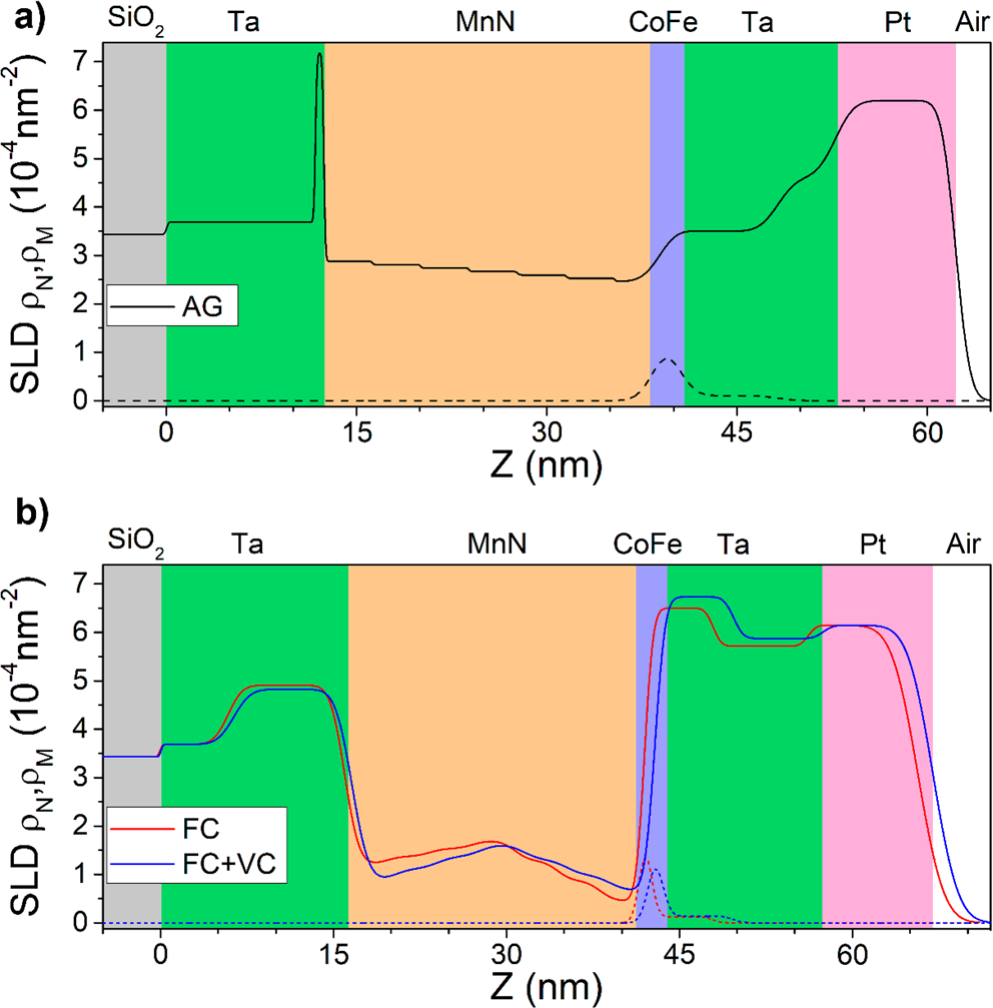
SLD depth profiles for (a) the AG state (black) and (b) the FC (red) and FC+VC (blue) states of Ta (10 nm)/MnN (30 nm)/Co_.7_Fe_0.3_ (1 nm)/Ta (10 nm)/Pt (10 nm). Solid lines represent the nuclear component of the SLD, and dashed lines represent the magnetic component of the SLD. The background colors indicate locations of various layers in the sample heterostructure.

Using PNR, the structural and magnetic depth profiles of the Ta (10 nm)/MnN (30 nm)/Co_0.7_Fe_0.3_ (1 nm)/Ta (10 nm)/Pt (10 nm) structure are probed in the AG, FC, and FC+VC states,^44,45^ as shown in Figure 4. An additional benefit of PNR for our structure is that small variations in N concentrations in the MnN layer should produce significant changes in the scattering length density (SLD). This is because Mn has a negative nuclear SLD (ρ_N_ = −2.98 × 10^–4^ nm^–2^), while MnN has a large positive nuclear SLD (ρ_N_ = 1.77 × 10^–4^ nm^–2^), leading to large contrast in ρ_N_ between stoichiometric MnN and Mn. Fits of the PNR data for each state using the chosen model are shown in Supporting Information (Figure S9), along with other excluded fitting models and a discussion of how the best model was chosen.

In the AG state (black curve in Figure 4a), the MnN layer is best modeled with a continuous gradient in ρ_N_. At the bottom Ta interface, MnN is modeled with a ρ_N_ of 2.88 × 10^–4^ nm^–2^ (uncertainty for all ρ_N_ < ±0.04 × 10^–4^ nm^–2^). At the top Co_0.7_Fe_0.3_ interface, ρ_N_ of MnN is modeled as 2.46 × 10^–4^ nm^–2^. This range is somewhat higher than the nominal MnN ρ_N_, indicating that the sample is N-rich, in agreement with the *c/a* ratio ∼1 observed in XRD. The gradient may be due to variations in N_2_ during sputtering or an intrinsic property of the MnN thickness, as observed in similar MnN heterostructures.^38^ The ρ_N_ for Ta and Pt in the AG state match closely with expected values, but the top region of the Ta seed and capping layers exhibit increased ρ_N_. Deviation near the Ta capping layer is understandable, as the samples were exposed to air before depositing a Pt layer, indicating the formation of TaO_*x*_ with a ρ_N_ of 4.61 × 10^–4^ nm^–2^ and a layer thickness of 4.5 nm, as stoichiometric Ta_2_O_5_ has a calculated ρ_N_ of 4.79 × 10^–4^ nm^–2^. The sharp increase in the Ta seed layer to ρ_N_ = 7.17 × 10^–4^ nm^–2^ at the Ta/MnN interface is likely due to exposure to N_2_ plasma during deposition, leading to N inclusion in the layer. This value of ρ_N_ is slightly above the nominal value of TaN (ρ_N_ = 6.89 × 10^–4^ nm^–2^), which may be an indication of an excess of N or intermixing of Mn into this region, as seen in HAADF-STEM. The magnetic component of the SLD, ρ_M_, is indicated by the dashed black line in Figure 4a. The only significant magnetic contribution is from the Co_0.7_Fe_0.3_ layer, though the model suggests a small magnetic inclusion in the top Ta layer, likely from Co, Fe, and Ta interdiffusion also observed by HAADF-STEM around the top Co_0.7_Fe_0.3_/Ta interface.

In the FC state (red curve in Figure 4b), significant changes in layer thickness and ρ_N_ are observed. In the MnN layer, ρ_N_ decreases overall and is sufficiently modeled by two linear gradients decreasing from the center of the MnN layer. The values at the bottom, middle and top regions of the MnN layer are all below the nominal MnN ρ_N_, with ρ_N,Bottom_ = 1.21 × 10^–4^ nm^–2^, ρ_N,Middle_ = 1.69 × 10^–4^ nm^–2^, ρ_N,Top_ = 0.45 × 10^–4^ nm^–2^, respectively. This depth profile indicates a significant reduction in N concentration in this layer, in agreement with HAADF-STEM, EELS, and XRD, and has an associated reduction in MnN thickness of 1.3 nm. This model indicates the direction of N diffusion out of MnN is toward both the Ta seed and capping layer (including the TaO_*x*_), as evidenced by the significant increase in ρ_N_. Interestingly, the Ta seed layer seems to have a lower N concentration than the Ta capping layer, with ρ_N_ = 4.91 × 10^–4^ nm^–2^ and ρ_N_ = 6.50 × 10^–4^ nm^–2^, respectively. The cause of this asymmetric N diffusion is unclear, but it may indicate that the intermixed FM layer plays a role in catalyzing N diffusion in the system or that the TaN interface on the Ta seed layer may act as a N diffusion barrier.^36^ As N diffuses into Ta, there also seems to be a significant increase in thickness of both Ta layers, with the seed layer increasing by 3.6 nm and the capping layer increasing by 0.8 nm, which may be explained by the lower density of TaN compared to Ta. *ρ*_M_ in the FC state decreases in the Co_0.7_Fe_0.3_ layer from 5.89 × 10^–4^ nm^–2^ in the AG state to 4.54 × 10^–4^ nm^–2^, which is consistent with the observed decrease in *m*_s_.

In the FC+VC state, PNR provides insight into the effect of applying an electric field and the corresponding increase in *H*_E_ (Figure 4b, blue curve). First, a decrease in ρ_N_ is observed in the Ta seed layer (ρ_N_ = 4.82 × 10^–4^ nm^–2^) and in the bottom half of the MnN layer ρ_N,Bottom_ = 0.87 × 10^–4^ nm^–2^; ρ_N,Middle_ = 1.62 × 10^–4^ nm^–2^), which corresponds to a decrease in N concentration. An increase in ρ_N_ relative to the FC state is also observed in the top half of the MnN layer (ρ_N,Top_ = 0.67 × 10^–4^ nm^–2^) and the Ta capping layer (ρ_N_ = 6.73 × 10^–4^ nm^–2^), indicating the net motion of N under bias is +*Z*, toward the top contact. The change in ρ_N_ in the MnN and Ta layers is statistically significant, as the 95% confidence intervals of the modeled ρ_N_ do not have any overlap in the bottom of MnN (95%CI_FC_ = 1.19–1.25 × 10^–4^nm^–2^; 95%CI_FC__*+*VC_ = 0.85–0.90 × 10^–4^ nm^–2^), the top portion of MnN (95%CI_FC_ = 0.41–0.49 × 10^–4^ nm^–2^; 95%CI_FC__*+*VC_ = 0.64–0.70 × 10^–4^ nm^–2^), the Ta seed layer (95%CI_FC_ = 4.88–4.94 × 10^–4^ nm^–2^; 95%CI_FC__+VC_ = 4.79–4.85 × 10^–4^ nm^–2^), or the Ta capping layer (95%CI_FC_ = 6.46–6.54 × 10^–4^ nm^–2^; 95%CI_FC__+VC_ = 6.70–6.77 × 10^–4^ nm^–2^). Small changes in layer thicknesses (0.2–0.4 nm for Ta and MnN layers), leading to the total thickness offset in Figure 4b, and insignificant changes in magnetization from the FC to FC+VC were also seen in this model.

## Discussion

The interesting electric-field enhancement of the EB can be understood by nitrogen ionic migration. In the AG state, N is present in the top region of the Ta seed layer, which may occur due to spontaneous gettering of N by Ta from the MnN layer. Using the stoichiometries for tabulated thermodynamic properties of Ta and θ-phase MnN (Mn_6_N_5_), the reaction would follow eq 1 below. The spontaneous gettering of N from MnN by Ta is supported by the calculated Gibbs free energy of −132.6 kJ/mol and a calculated enthalpy of formation of −137.3 kJ/mol,^46−48^ indicating the interfacial reaction would be both spontaneous and exothermic.
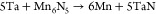
1

Furthermore, during the FC process, N diffusion is enhanced by the elevated temperatures and N moves to both the Ta capping layer and seed layers since the direction of the reaction remains the same, as seen in EELS and PNR. This subsequently leads to both induced EB while cooling the sample in a magnetic field and a reduction in N content in the MnN layer. STEM and magnetometry both suggest that intermixing at the MnN/Co_0.7_Fe_0.3_/Ta interfaces occur in the AG state, and exposure to elevated temperatures during FC leads to further intermixing.

After the FC+VC process, PNR’s depth resolution, particularly its sensitivity to N, indicates that N indeed migrates in the structure. The direction of this motion is + Z under a positive bias, with N moving toward the top of the MnN layer, as well as into the Ta capping layer. No difference in the amount of Mn_3_N_2_ is seen by HAADF-STEM or XRD after gating, suggesting this N increase is within MnN itself. The increase in *H*_E_ can be attributed to this N motion, as N content increases in MnN near the Co_0.7_Fe_0.3_/MnN interface after gating, which is supported by literature where increased exchange bias with N content is well observed in MnN systems due to increases in the interfacial exchange constant, *J*_ex_, with increasing N content.^35,36,38,39^ This effect may be considered as equivalently an increase in the effective AF layer thickness, consistent with a 0.2 nm increase in the MnN layer thickness observed in PNR, that helps to provide a stronger pinning of the FM, thus a larger EB. Another possible source of *H*_E_ enhancement can be attributed to the observed N diffusion into the Ta capping layer under biasing. It is likely that defects are introduced at the top MnN/Co_0.7_Fe_0.3_ interface as a result of the nitrogen migration, leading to changes in pinned uncompensated AF moments, which sensitively influences *H*_E_.^2,4,49−52^ This is highlighted by the observation of significantly different EB values for this structure compared to the reference sample without a Ta capping layer (Figure S2). Though it could only be demonstrated for the FC state since the reference sample had essentially no EB, it does indicate that N moving to the Ta capping layer during the field cooling process is an important component of the observed EB. Driving more N to this Ta layer in the FC+VC states could then also play a significant role in the EB enhancement. Even a small amount of N migration may cause sufficient modification of interface to result in a significant change in EB. No significant contributions to EB enhancement are expected to be caused by Mn_3_N_2_ in the MnN layer, as the FC process occurred well below the *T*_N_ of η-Mn_3_N_2_ and thus is not expected to alter the interfacial FM/AF coupling.

In the FC+VC-VC state, the bias is reversed across the sample and N migration is expected to be from the Ta capping layer and upper half of MnN toward the lower half of MnN and the Ta seed layer. The decrease in EB under negative biasing, along with an increase in EB following a subsequent positive biasing indicates the potential for reversible control of EB with electric fields. While the data shown in Figure S1 is representative of the continued gating trend in most samples of similar structure, it should be noted that the first cycle (positive and negative bias) trend is consistent across all samples and that deviations have been seen in a small number of samples after the first cycle. These deviations in trend are believed to be a result of the sample structure, with two relatively symmetric N reservoirs (Ta layers partially converted to TaN) around MnN. These layers provide a source of N regardless of the gating direction, which will be addressed by future studies.

The requirement of longer gating times to achieve full reversibility under negative biasing can be understood from the thermodynamic properties of the system. First, the negative Gibbs energy of the reaction in eq 1 indicates that the formation of TaN is preferred, which would require less energy to drive the reaction forward. Conversely, the energy required to reverse the direction of N migration will be greater due to this same factor. Additionally, even as MnN decomposes to Mn_3_N_2_ as seen in HAADF-STEM, the calculated Gibbs free energy is −212.6 kJ/mol for the reaction shown in eq 2,^47,48^ indicating even as N is lost, the remaining Ta will preferentially form TaN over the reverse reaction.
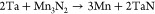
2

## Conclusions

In summary, significant nitrogen-based magneto-ionic enhancement of exchange bias has been observed in Ta/MnN/Co_0.7_Fe_0.3_/Ta heterostructures, which can be electrically manipulated. A comprehensive set of studies using magnetometry, HAADF-STEM, EELS, and PNR has enabled probing of structural, magnetic, and N concentration changes across the structure under different sample conditions. When samples are field cooled, a clear N migration out of the MnN layer and into the Ta layers is evident, and the formation of Mn_3_N_2_ grains occur. This field-cooling step leads to a significant exchange bias. Upon positive voltage biasing, *H*_E_ increases by 19% at 10 K and 5% at room temperature. This enhancement corresponds to both N migration in the + Z direction into the top half of the AF MnN layer, as well as into the Ta capping layer. It is both the increased N content in MnN and likely changes to the pinned uncompensated AF spins at the interface that contribute to the enhancement of EB. Reverse biasing for a longer duration leads to the recovery of the initial EB; a subsequent positive bias enhances EB yet again, indicating that this electric control of EB may persist beyond one cycle. These results demonstrate the potential for electrical manipulation of exchange bias via the magneto-ionic handle. The modulation achieved using the nitrogen ions has potential applications in low energy-dissipation nanoelectronics.

## Methods

### Sample Synthesis

Thin films of Ta seed (10 nm)/MnN (30 nm)/Co_0.7_Fe_0.3_ (1 nm)/Ta (10 nm)/Pd or Pt cap (10 nm) (bottom to top structure) were grown on thermally oxidized p-type Si substrates (SiO_2_ = 285 nm) following a standard cleaning procedure in acetone, isopropanol, and deionized water. A shadow mask was used to pattern the samples into 5 mm × 5 mm arrays. All sputtering depositions were performed in a chamber with a base pressure of <6 × 10^–6^ Pa, and a working pressure of 0.33 Pa. The 10 nm Ta seed layer was first grown by direct current (DC) magnetron sputtering. The 30 nm MnN was then radio frequency (RF) reactively sputtered from an elemental Mn target with a 1:1 N_2_/Ar mixture.^35,39^ The 1 nm CoFe was grown onto MnN by either DC cosputtering from elemental Co and Fe targets or from a single Co_0.7_Fe_0.3_ composite target. In the co-sputtering case, deposition power was calibrated to achieve a 70:30 ratio of Co/Fe. Finally, the samples were capped with 10 nm Ta, removed from the chamber (<1 h air exposure), and reintroduced to the chamber for a 10 nm Pd or Pt top electrode deposition. A reference sample without a Ta capping layer for magnetometry comparison was also prepared with the same conditions (10 nm)/MnN (30 nm)/Co_0.7_Fe_0.3_ (1 nm)/Ta (10 nm)/Pd or Pt cap (10 nm).

### Magnetic Measurements

Magnetic characterizations were conducted using a superconducting quantum interference device (SQUID) magnetometer (Quantum Design MPMS3). To establish exchange bias, the sample was heated inside the SQUID to 700 K at a rate of 50 K/min, above the Néel temperature of MnN (*T*_N_ ∼ 660 K).^41,42^ Once this temperature was reached, a 6.5 kOe in-plane magnetic field was applied and held for 1 min. Subsequently, the sample was cooled to 300 or 10 K in this field at a cooling rate of 50 K/min. Hysteresis loops were measured with a saturation magnetic field of 20 kOe for samples in the as-grown (AG), field cooled (FC), and subsequent voltage conditioned (VC) states (FC+VC and FC+VC-VC). Here, FC+VC refers to a positive gating of 20 V for 1 h after field cooling, using a Keithley 2280S Precision Measurement DC supply, which was also used to monitor any potential oxide breakdown. The FC+VC-VC refers to the subsequent negative biasing for 1 h after the initial positive gating. After field cooling each sample, magnetic field treatments were used to decrease the field training effect observed in the samples to below the measurement error (details in Supporting Information). The reference sample without the Ta capping layer was also field cooled and measured under the same conditions at 300 K for comparison.

### X-ray Diffraction

Structural characterization by X-ray diffraction was performed using a Malvern-Panalytical X’Pert3 MRD system with Cu K*_α_* radiation in both θ–2θ and grazing incidence (GIXRD) configurations. The θ–2θ scans were performed over a 2θ range of 20–130° using a PixCel line detector with a step size of 0.02° and total integration time of 1000 s. GIXRD scans used an incidence angle of 0.5°, a Xe proportional detector (point detector) with a step size of 0.05°, and a total integration time of 60 s over a range of 30–70° in 2θ.

### Neutron Scattering

Polarized neutron reflectometry measurements were carried out at NIST Center for Neutron Research on the Polarized Beam Reflectometer. Measurements were taken at room temperature with a 15 kOe magnetic field applied in-plane along the field-cooling axis of the samples. The neutron beam was polarized parallel (+) or antiparallel (−) to the magnetic field, and nonspin-flip specular reflectivities (R_++_ and R_–_) were measured with respect to wave vector transfer, *Q*. The REDUCTUS and Refl1D software packages were used to reduce and fit the data, respectively.^44,45^ Error bars were determined with a Markov chain Monte Carlo method using the BUMPS software package.

### Electron Microscopy

Electron microscopy studies were performed at the NIST Materials Measurement Laboratory. Electron transparent cross-sectional samples were prepared with an FEI Nova NanoLab 600 DualBeam (SEM/FIB). An FEI Titan 80–300 probe-corrected STEM/TEM microscope operating at 300 keV was employed to conduct atomic-resolution HAADF-STEM imaging and EELS analysis.

## Supporting Information Available

Additional experimental details, methods, and analysis relating to the gating response, magnetometry, and PNR (PDF).
