# Supplementary material for: Nitrogen-Based Magneto-ionic Manipulation of Exchange Bias in CoFe/MnN Heterostructures
Source: ACS Nano. 2023 Mar 30;17(7):6745–53. doi: 10.1021/acsnano.2c12702 (PMC10950296; doi:10.1021/acsnano.2c12702)
Supplement: Supplementary file 1 — nn2c12702_si_001.pdf [file nn2c12702_si_001.pdf]

Nitrogen-Based Magneto-Ionic Manipulation of Exchange Bias in CoFe/MnN Heterostructures

Christopher J. Jensen[1], Alberto Quintana[1], Patrick Quarterman[2], Alexander J. Grutter[2], Purnima P. Balakrishnan[2], Huairuo Zhang[3,4], Albert V. Davydov[4], Xixiang Zhang,[5] and Kai Liu[1,*]

[1]*Physics Department, Georgetown University, Washington, DC 20057, USA*
[2]*NIST Center for Neutron Research, NCNR, National Institute of Standards and Technology, Gaithersburg, MD 20899, USA*
[3]*Theiss Research, Inc. La Jolla, California 92037, USA*
[4]*NIST Materials Measurement Laboratory, National Institute of Standards and Technology, Gaithersburg, MD 20899, USA*
[5]*King Abdullah University of Science & Technology, Thuwal 23955-6900, Saudi Arabia*

Gating Modulation of Exchange Bias

Repeated voltage gating was performed on the sample shown in the main text magnetometry result (Fig. 1). The sample was first subjected to a positive bias for 1 hour, which led to the enhancement of exchange bias, as described in the main text. Fig. S1 shows the exchange field, H_E, for this as well as subsequent gating of -20V for 1 hour, a repeated -20V for 1 hour, and a final +20V for 5 hours. H_E is approximately revered after the second negative biasing, reaching a value of 620 Oe, and positively gating for 5 hours led to an increase of H_E to 628 Oe.

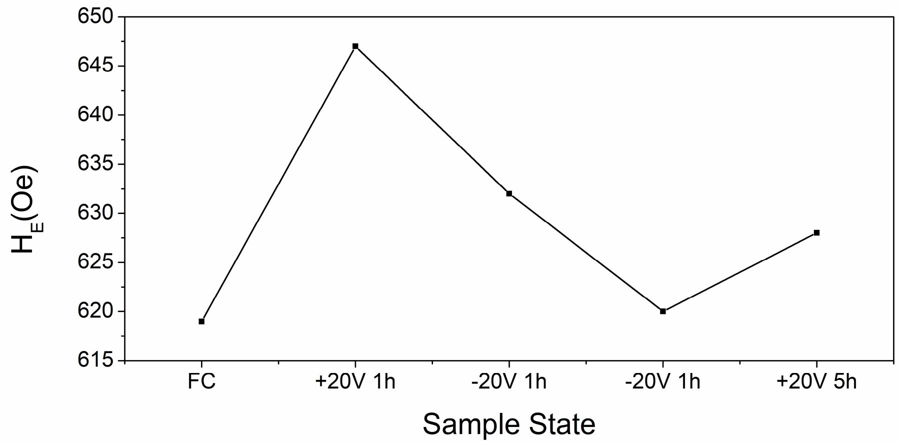

Figure S1. Exchange bias gating trends of the Ta (10 nm)/MnN (30 nm)/CoFe (1 nm)/Ta (10 nm)/Pd (10 nm) sample. The *x*-axis denotes the gating/preparation step performed before the corresponding H_E value was measured, with each step being sequentially performed on the same sample.

Capping Layer Dependence of Exchange Bias

A reference sample was grown of the structure in the main text, but without a Ta capping layer before the Pd contact was deposited. Remarkable differences in H_E was observed, as shown in Fig. S2, with the reference sample exhibiting almost no EB. In addition, there is little magnetic coercivity seen. This indicated that Ta plays a significant role in establishing EB for the conditions used.

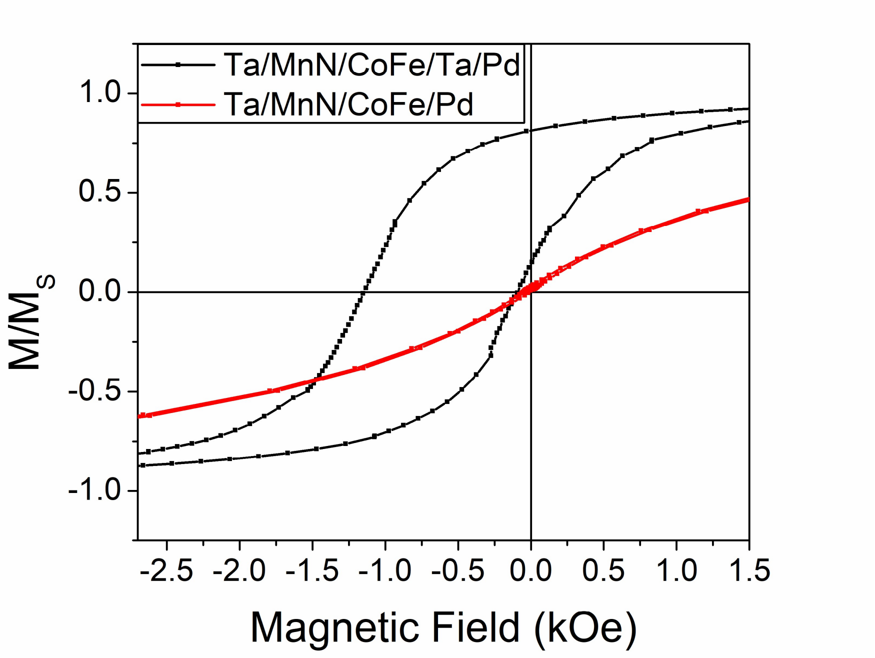

Figure S2. The FC state hysteresis loops at 300 K of the Ta (10 nm)/MnN (30 nm)/CoFe (1 nm)/Ta (10 nm)/Pd (10 nm) structure compared to a reference sample without a Ta capping layer of Ta (10 nm)/MnN (30 nm)/CoFe (1 nm)/Pd (10 nm). The magnetic moment is normalized to the saturation moment of each sample.

X-Ray Diffraction

X-ray diffraction θ-2θ scans over an extended range of 20˚-110˚ in 2θ are shown in Figure S3 for each state of the Ta (10 nm)/ MnN (30 nm)/ $Co_{0.7}Fe_{0.3}$ (1 nm)/ Pd (10 nm) heterostructure. The same scans are presented in the main text in Fig. 2b over the 2θ range of 40.0˚-45.5˚ to highlight changes in the MnN layer. In addition, a reference scan on an identical thermally oxidized Si substrate is included, measured with the same beam configuration as the samples.

The MnN (002)/(200) and Mn_3N_2 (006)/(200) peak in Fig. 2b are discussed in the main text of this paper, but to confirm (002)/(200) texture in MnN all other peaks must be identified. Peaks for each scan in the 46˚-80˚ region can all be attributed to the substrate and background of the instrument, as each peak present is also in the substrate reference scan. Additional peaks were identified for the AG, FC, and FC+VC states at 37.3˚, 40.3˚, and 104.9˚. Tabulated peaks for tetragonal Ta have peaks corresponding to the (330) reflection at 37.4˚ and for the (411) reflection at 40.2˚ (PDF 00-025-1280). The 104.9˚ peak corresponds to the (400) Pd tabulated peak at 104.8˚ (PDF 00-046-1043). An additional peak is present in the FC and FC+VC states at 93.4˚, which corresponds to the (004)/(400) reflections of θ-phase MnN at 93.5˚[1] and the (400) reflection of η-phase Mn_3N_2 at 94.4˚ (PDF 00-001-1158). One final peak is present in the FC+VC state, located at 33.0˚, which due to the sharpness of the peak is attributed to a forbidden reflection of Si. The (002)/(200) texture of MnN in the AG state, as well as the c-axis texture corresponding to the convoluted peaks of (002)/(200) MnN and (006)/(200) Mn_3N_2 in the FC and FC+VC states are supported by no other MnN or Mn_3N_2 reflections being present in the θ-2θ scan data.

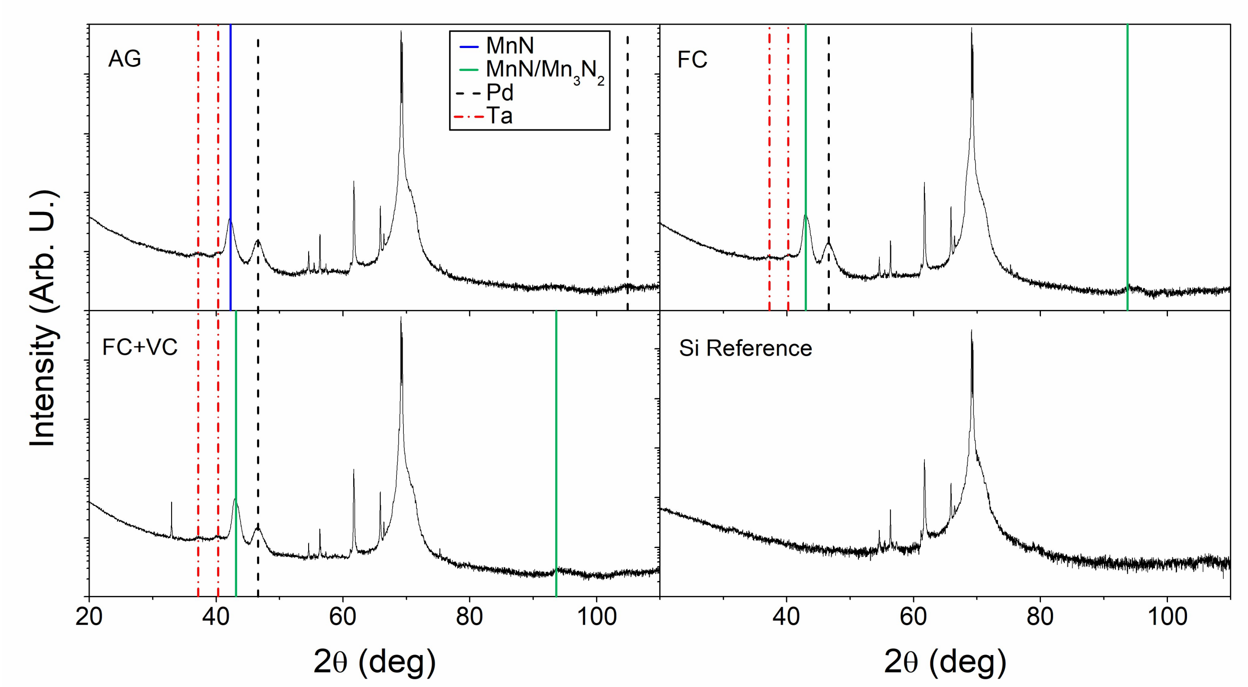

Figure S3. Full range X-ray diffraction θ-2θ scans of the AG, FC, and FC+VC states of Ta (10 nm)/MnN (30 nm)/CoFe (1 nm)/Pd (10 nm) sample and a reference Si substrate. Each plot is on a log scale in intensity and has MnN, MnN/Mn_3N_2, Pd, and Ta peaks labeled. Unlabeled peaks correspond to Si and detector background reflections.

STEM-EELS

STEM-EELS line-scans profiles were taken for cross-sections of the Ta/MnN/$Co_{0.7}Fe_{0.3}$/Ta/Pd structure in the AG, FC, and FC+VC states. The AG state (Fig. S4a) shows a higher relative concentration of N in the MnN layer compared to both Ta layers. After the structure is exposed to 700 K during the field cooling process (Fig. S4b), the relative concentration of N in the MnN layer and Ta layers are approximately equal. This suggests that N moves from the MnN layer and into both the Ta seed and capping layer.

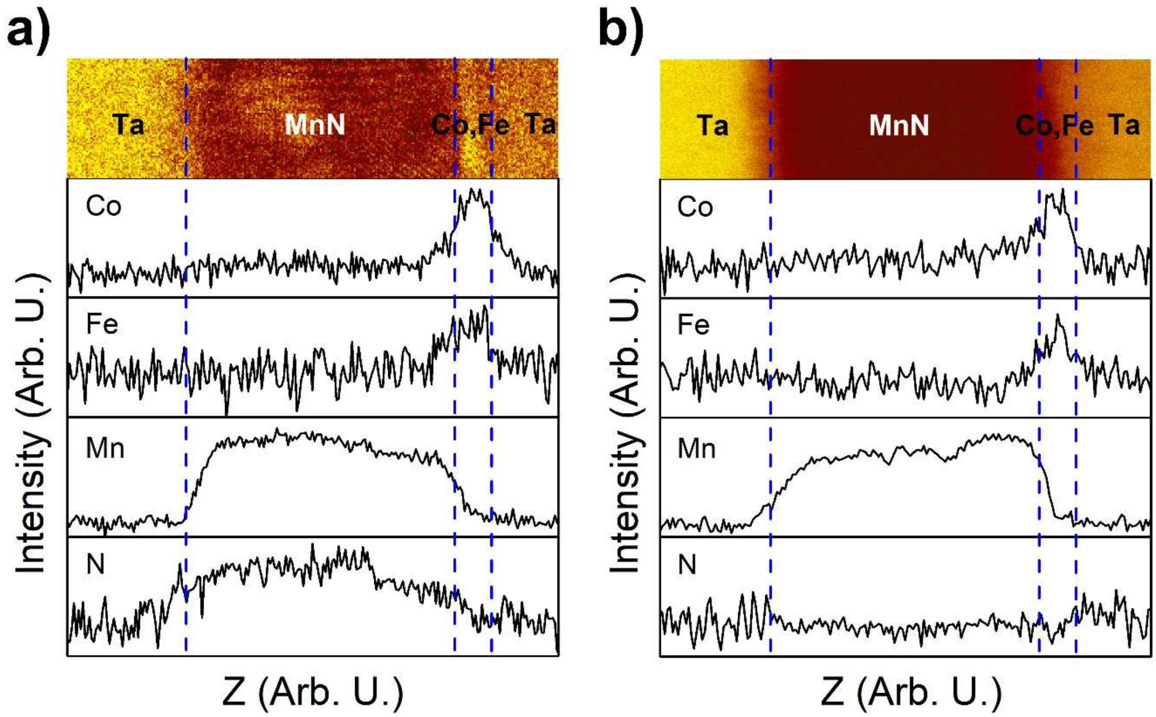

Figure S4. STEM-EELS line-scan profiles for the Ta (10 nm)/MnN (15 nm)/$Co_{.7}Fe_{0.3}$ (1 nm)/Ta (10 nm)/Pd (10 nm) structure in the a) AG and b) FC states. The top panels show cross sections of the HAADF-STEM images, and each panel is labeled with the representative element. Dashed black lines are used to highlight the interfaces.

Polarized Neutron Reflectometry Modeling

Polarized Neutron Reflectometry (PNR) measurements were conducted at NIST Center for Neutron Research. Reduction and fitting of the data were performed in the REDUCTUS and Refl1D software packages.[2-3] While these packages offer powerful fitting algorithms, model optimization remains a challenge when performing simultaneous refinement off multiple datasets with linked models and a wide parameter space, as is often the case for magneto-ionic systems. Similarly, solution degeneracy means that more than one model is likely to sufficiently describe a reflectivity dataset. Thus, models must be chosen such that the fitting is not over or under parameterized while enforcing physically reasonable boundary conditions. To achieve this, we chose to start with the simplest models and work toward more complex models, ultimately choosing the best model that was supported by additional measurements on the heterostructure, such as electron microscopy. For each model, the AG, FC, and FC+VC states were fit simultaneously in order to enforce parameters uniformity for layers, such as Si and SiO_2, across different sample states that were expected to remain unchanged. By fitting in this way, we avoid unphysical solutions in which layers not participating in voltage-driven electrochemical reactions change between field conditions, while simultaneously achieving improved statistical certainty on the derived parameters. In this section, we will describe the models that were excluded and how we arrived at our chosen model presented in the main text of the paper.

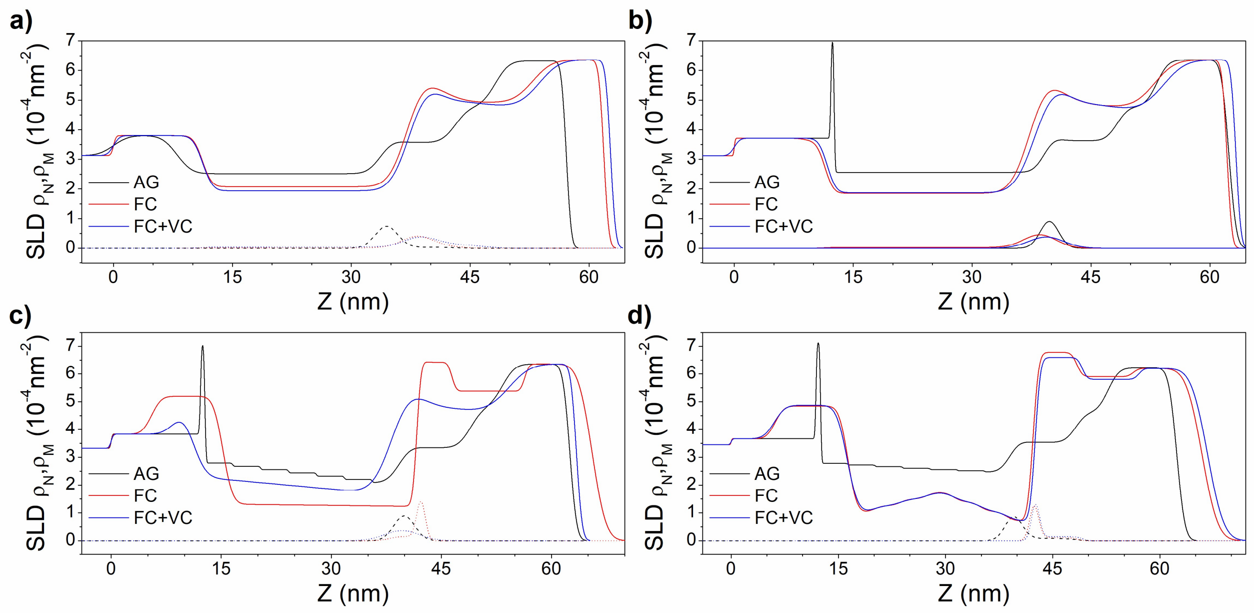

Figure S5. SLD depth profiles for the AG state (black), FC state (red), and FC+VC state (blue) of Ta (10 nm)/MnN (30 nm)/$Co_{0.7}Fe_{0.3}$ (1 nm)/Ta (10 nm)/Pt (10 nm). The panels correspond to a model using a) constant nuclear components of the SLD, b) constant nuclear component of the SLD and inclusion of interfacial N in the Ta seed layer, c) linear gradients in the nuclear component of the SLD, and d) linear gradients in the nuclear components of the SLD in the MnN layer that were allowed to vary in two different regions.

Constant SLD Model

For the simplest model, each layer in the heterostructure was fit using a constant nuclear, ρ_N, and/or magnetic, ρ_M, component of the scattering length density, SLD. The layers included in the model were Ta, MnN, $Co_{0.7}Fe_{0.3}$, Ta, TaO_x, and Pt from bottom to top of the structure, respectively. TaO_x was included due to the sample being removed from the deposition chamber between the Ta and Pt growth, which likely caused oxidation. Layer thicknesses and roughness were given initial values estimated from growth parameters, and ρ_N and ρ_M were calculated from density and saturation magnetization for each layer.

The SLD profile for this model is shown in Fig. S5a, where a variation in thickness from the AG state to FC and FC+VC states is indicated. Qualitative analysis of the fittings, shown in Fig. S6, can be used to immediately see that this model does not fit the data well between 0.7 to 1.2 nm^{-1} in Q for the AG state (Fig. S6a). Additionally, poor fitting is seen for both the FC (Fig. S6c) and FC+VC (Fig. S6e) conditioned states. Quantitatively, this model has the highest χ^2 for the AG (χ^2 = 3.67) and FC state (χ^2 = 3.55) of any model. Spin asymmetry $[SA = (R_{++} - R_{--})/(R_{++} + R_{--})]$ is calculated for both the measured data and simulated fits and plotted for the AG (Fig S6b), FC (Fig. S6d), and FC+VC (Fig. S6f) states. SA arises from reflectivity differences in the R_{++} and R_{--} reflectivity cross sections when interacting with magnetic materials, but also contains structural information. The poor fits in the 0.7 to 1.2 nm^{-1} range of Q for SA additionally highlight the ineffectiveness of this model. This model was excluded for these reasons.

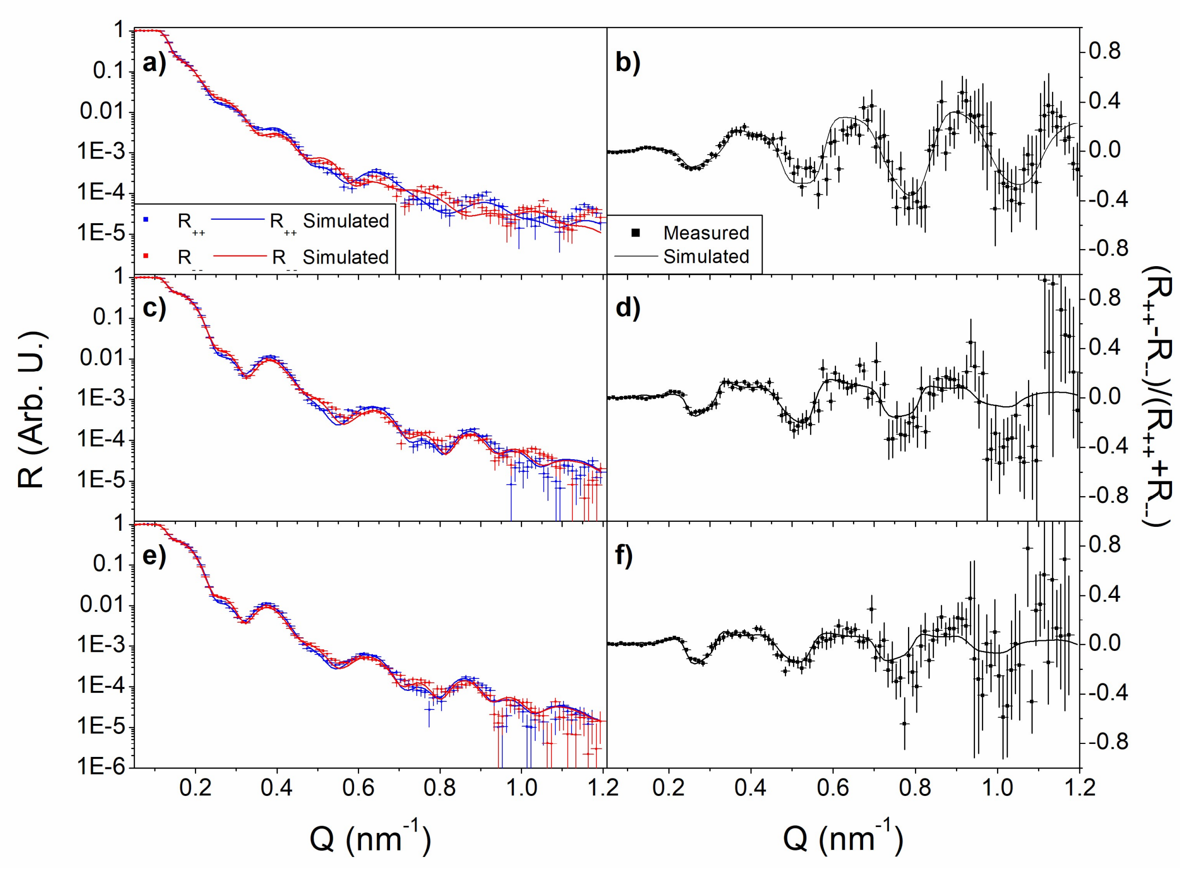

Figure S6. a,c,e) Fits of the reflectivity data and b, d, f) calculated spin asymmetry using the sample model of constant nuclear SLD values in each layer of Ta (10 nm)/MnN (30 nm)/$Co_{.70}Fe_{0.3}$ (1 nm)/Ta (10 nm)/Pt (10 nm). Reflectivity fits and spin asymmetry calculations are presented for the a,b) AG, c,d) FC and e,f) FC+VC states, respectively.

Constant SLD + TaN Model

To improve on the previous model, a TaN layer was added to the model at the Ta/MnN interface. This layer was observed in the PNR analysis of similar samples by Quarterman et al.[1] and is expected to form due to N_2 exposure to Ta during sputter growth of MnN. This layer was again fit with a constant ρ_N. All other parameters follow with the previous model. Fig. S5b shows the modeled SLD profile corresponding to this model, with the only significant change seen in the AG state, where a sharp peak with increase ρ_N is present at the Ta/MnN interface, corresponding to TaN ($\rho_N(Ta) = 3.82\ x\ 10^{-4} nm^{-2};\ \rho_N(TaN) = 7.19\ x\ 10^{-4} nm^{-2}$) .

From the fitted PNR data, shown in Fig. S7, an improvement is seen in the AG state fit (Fig. S7a), especially in the 0.7 to 1.0 nm^{-1} range in Q, which is also reflected in the SA seen in Fig. S7b. Additionally, χ^2 slightly improves for the AG ($\chi^2 = 3.50$) and FC state ($\chi^2 = 3.35$), though the fits for the FC (Fig. S7c) and FC+VC (Fig. S7e) states qualitatively do not match the data well. SA for the FC (Fig. S7d) and FC+VC (Fig. S7f) show little to no improvement in the 0.7 to 1.2 nm^{-1} range in Q, and the SA fit is qualitatively worse for the FC+VC state, especially around 0.3 nm^{-1}. Because of the poor fits for the FC and FC+VC states, this model was excluded.

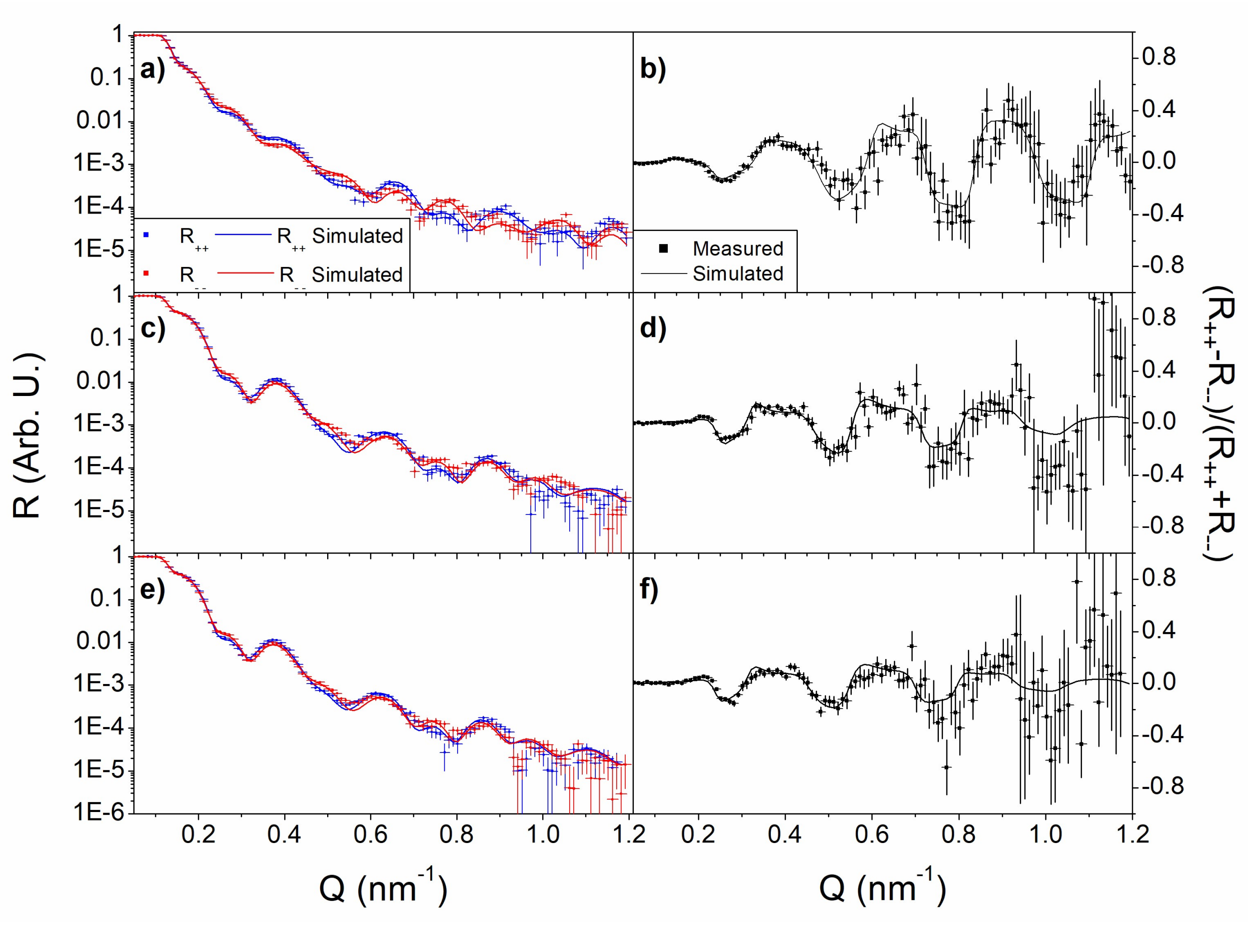

Figure S7. a,c,e) Fits of the reflectivity data and b, d, f) calculated spin asymmetry using the sample model of constant nuclear SLD values in each layer of Ta (10 nm)/MnN (30 nm)/$Co_{.7}Fe_{0.3}$ (1 nm)/Ta (10 nm)/Pt (10 nm) and allowing interfacial N at the Ta/MnN seed layer interface. Reflectivity fits and spin asymmetry calculations are presented for the a,b) AG, c,d) FC and e,f) FC+VC states, respectively.

MnN Linear Gradient Model

As it became evident that constant ρ_N values were not sufficient to model the PNR data, the next logical choice was to allow linear gradients to be fit across layers where there may be structural and/or magnetic variations. To avoid introducing too many parameters at once, a linear gradient was only added to the MnN layer in the structure since N variations within this layer may be significant, especially between the AG to FC state as seen in the STEM-EELS measurements shown in Fig. S4. To achieve this gradient, MnN was broken into 7 separate layers, with the top-most and bottom-most layers being fit. MnN layers 2-6 were calculated to create a linear gradient with equal step sizes in ρ_N from the top to bottom layers. This linear gradient was allowed to vary such that ρ_N on the top region of MnN could be higher, lower, or equal to the bottom region. All other parameters in this model were kept the same as the previous model, still allowing a TaN layer to be present.

Fig. S5c shows the fit SLD profiles for each state of the sample for this model. A decreasing linear gradient in ρ_N from the bottom to the top of the MnN layer is present in the AG and FC+VC states, while the FC state has a relatively constant ρ_N. TaN again seems to be present at the Ta/MnN interface in the AG state, indicated by the sharp increase in ρ_N, and a TaN layer is also present in the fits for the FC and FC+VC conditioned states. Additional interface roughness changes are seen in the SLD profiles, where the roughness increase across most multilayer interfaces from the FC to FC+VC states. Large structural changes such as these are not expected following voltage biasing, so this model does not fit the physical picture of the sample well. Additionally, the fit PNR data shown in Fig. S8 has improvements qualitatively and in χ^2 for the AG (Fig. S8a; χ^2 = 2.64) and FC state (Fig. S8c; χ^2 = 3.13), but the fit for the FC+VC (Fig. S8e) state worsens. A similar trend is also seen in the SA where the FC state (Fig. S8d) has a closer match between

simulated model and experimental data at higher Q values, and the FC+VC state (Fig. S8f) worsens overall. Here, we note that because of the linked parameters across all three models, the χ^2 variation of the fit to any given layer is not always intuitive. Rather, the software optimizes the overall integrated χ^2 for the linked model across all datasets. Thus, while a given model might fit one condition better, the overall fit across all conditions must be considered when evaluating which models to use. Because of the poor fit in the FC+VC state and non-physical interface roughness increases from the FC to FC+VC states, this model was excluded.

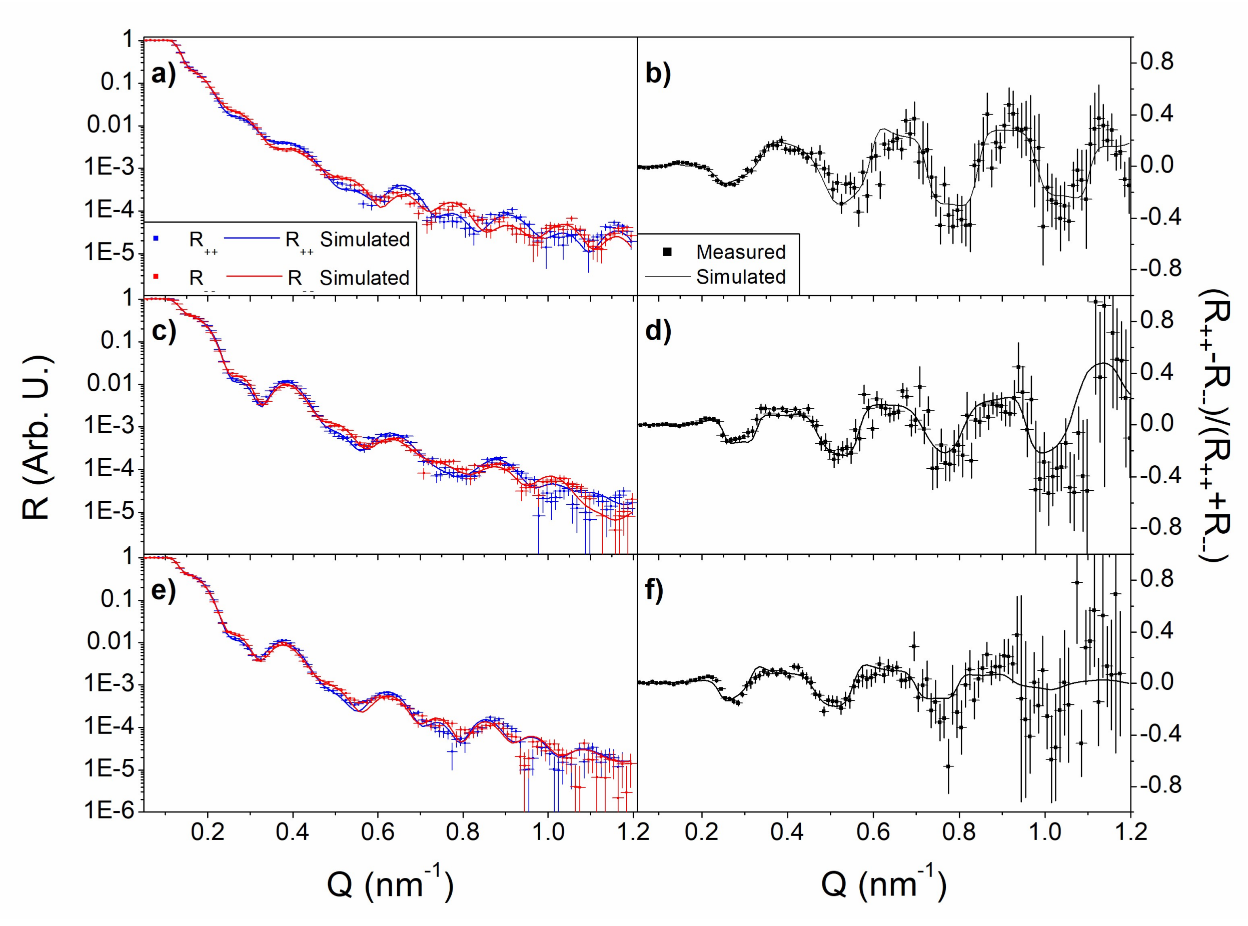

Figure S8. a,c,e) Fits of the reflectivity data and b,d,f) calculated spin asymmetry using the sample model of linear gradients in the MnN layer for the nuclear SLD values in of Ta (10 nm)/MnN (30 nm)/$Co_{.7}Fe_{0.3}$ (1 nm)/Ta (10 nm)/Pt (10 nm). Reflectivity fits and spin asymmetry calculations are presented for the a,b) AG, c,d) FC and e,f) FC+VC states, respectively.

Chosen Model - Multiple Linear Gradient

The chosen model for the PNR data came from the next step in modeling variation. For this, instead of a constant linear gradient across MnN, two separate linear gradients were allowed to be fit. This was chosen due to the Ta capping and seed layers, which are N getters, likely pulling N from both sides of the MnN layer. To achieve the gradients, the MnN layer was broken into the same 7 layers, but with the top-most, bottom-most, and middle layer being fit. Layers 2, 3, 5, and 6 are calculated to create equal size steps in ρ_N between the bottom to middle layer and the middle to top layer. All other layer parameters are the same as the previous model.

A detailed discussion of the fit SLD profiles for this model are presented in the main text and shown in Fig. 4. PNR data fits for each state are shown in Fig. S9. Significant qualitative and χ^2 improvements are seen for the AG (Fig. S9a; $\chi^2 = 1.74$), FC (Fig. S9c; $\chi^2 = 1.42$), and FC+VC state (Fig. S9e; $\chi^2 = 1.35$). Additionally, SA significantly improves in the 0.7-1.2 nm^{-1} range in Q for the FC+VC state (Fig. S9f), while the simulated fits for the AG (Fig. S9b) and FC (Fig. S9d) states still match the overall data SA profile well. Because of the good qualitative fits (especially in the 0.7-1.2 nm^{-1} range in Q), consistancy in physical meaning of ρ_N and ρ_M across the layers for each state, and best χ^2 values for any model used, this model was chosen as the most representative.

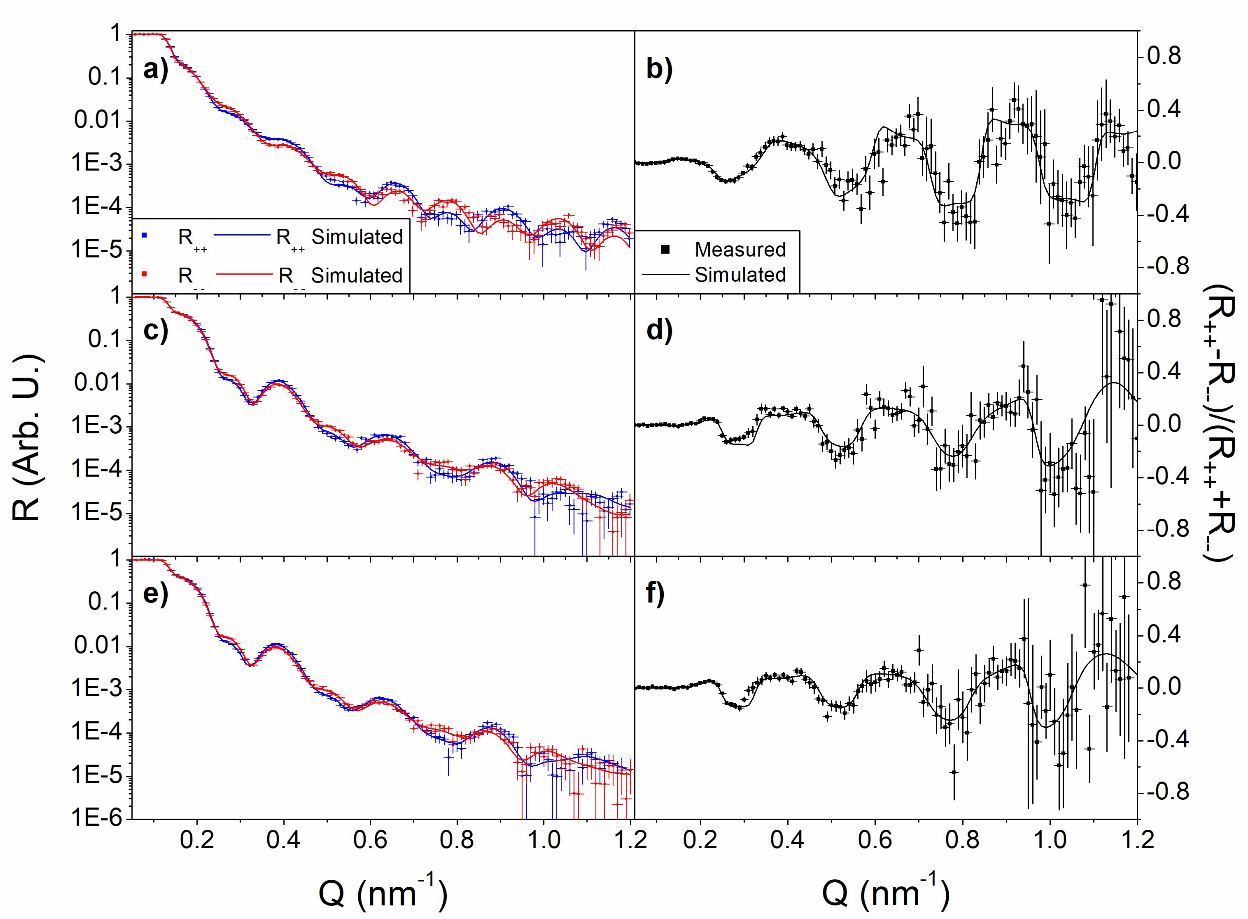

Figure S9. a,c,e) Fits of the reflectivity data and b,d,f) calculated spin asymmetry using the sample model of linear gradients in the MnN layer for the nuclear SLD values, with separate gradients able to exist in the top and bottom half of the layer in Ta (10 nm)/MnN (30 nm)/$Co_{.7}Fe_{0.3}$ (1 nm)/Ta (10 nm)/Pt (10 nm). Nuclear scattering length densities in the MnN layer for the FC and FC+VC states are set to be equal. Reflectivity fits and spin asymmetry calculations are presented for the a,b) AG, c,d) FC and e,f) FC+VC states, respectively.

Multiple Linear Gradient + Linked Sample State Model

The last model, with fit SLD profiles shown in Fig. S5d, was chosen to help determine if the ρ_N variation seen from the FC to FC+VC state in the previous model are significant. To do this, the exact parameters from the previous model were used, but with the ρ_N for the top, middle, and bottom regions of MnN forced to be equal between the FC and FC+VC states. If there was no significant difference in ρ_N between the two states, they should be fit with similar χ^2 values and qualitatively match when using an identical MnN layer.

Fig. S10 shows the fit PNR data for this model. χ^2 for each state increases, with the largest increase in the FC (χ^2 = 1.50) and FC+VC state (χ^2 = 1.43). While the differences in χ^2 and qualitative data fitting are not large, this model was excluded since it does not provide as good of a fit as the previous model. Additionally, this provides evidence that the ρ_N difference from the FC to FC+VC state are small but significant, with further evidence provided from non-overlapping 95% confidence intervals in MnN for the chosen model, as discussed in the main text.

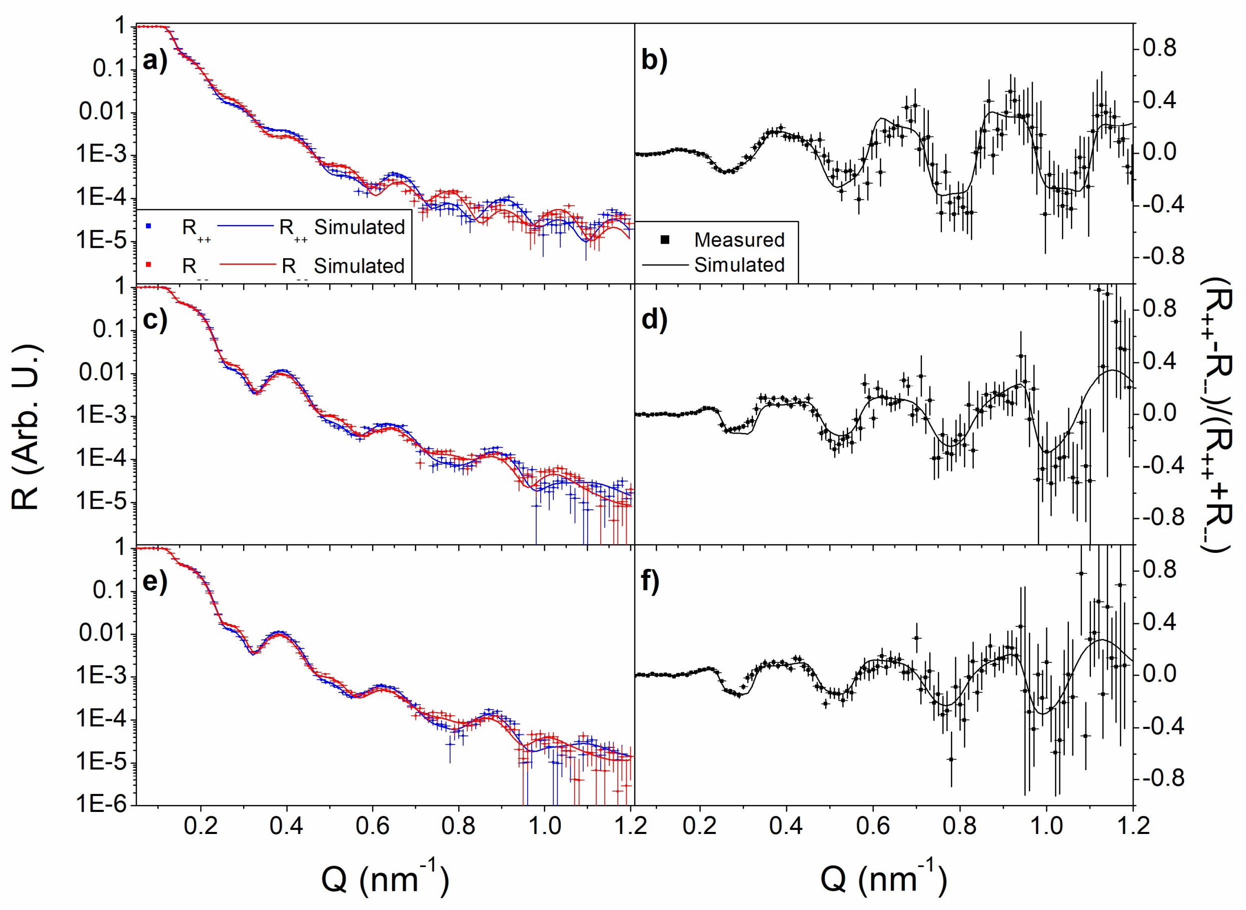

Figure S10. a,c,e) Fits of the reflectivity data and b,d,f) calculated spin asymmetry using the sample model of linear gradients in the MnN layer for the nuclear SLD values, with separate gradients able to exhist in the top and bottom half of the layer in Ta (10 nm)/MnN (30 nm)/$Co_{.7}Fe_{0.3}$ (1 nm)/Ta (10 nm)/Pt (10 nm). Reflectivity fits and spin asymmetry calculations are presented for the a,b) AG, c,d) FC and e,f) FC+VC states, respectively.

Field Training

To remove the field training effect often associated with exchange bias systems, after field cooling the samples at room temperature a 70 kOe field was applied for 5 minutes, followed by 10 cycles of 20 kOe to -20 kOe and holding the field at -20 kOe for an additional 30 minutes, which reduced the field training effect below measurement error. Exchange bias field, H_E, and coercive field, H_C, measured during repeated cycles after applying a 70 kOe field for 5 minutes are shown in Fig. S11. After 10 cycles, H_C has stabilized, and changes in H_E are almost an order of magnitude smaller (ΔH_E = -6 Oe) than after the first cycle (ΔH_E = -42 Oe). Holding the samples at -20 kOe for 30 minutes following cycling further decreases changes in repeated H_E measurements.

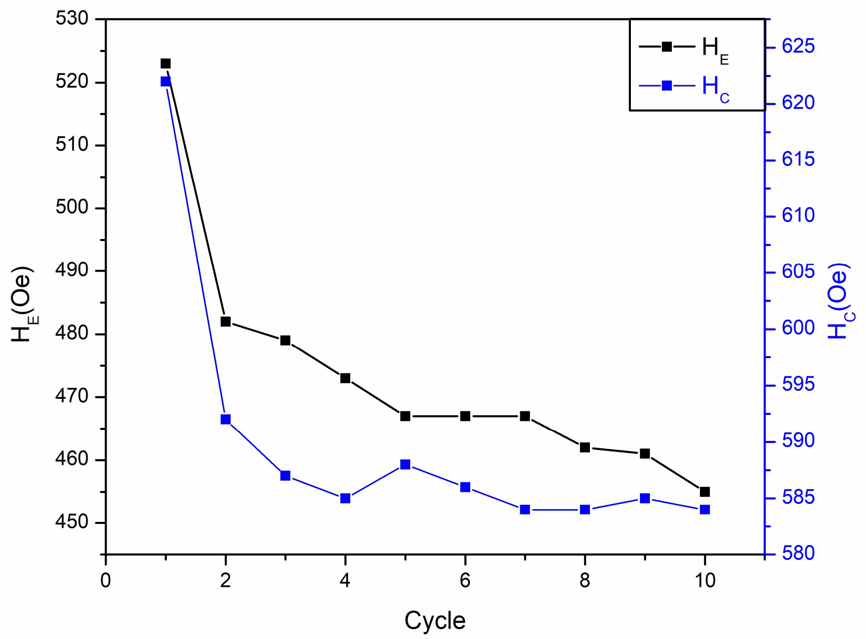

Figure S11. Exchange bias field, H_E, and coercive field, H_C, for the Ta (10 nm)/MnN (30 nm)/$Co_{.7}Fe_{0.3}$ (1 nm)/Ta (10 nm)/Pt (10 nm) measured repeatedly after applying a 7 T field for 5 minutes at room temperature.

References

1. Quarterman, P.; Hallsteinsen, I.; Dunz, M.; Meinert, M.; Arenholz, E.; Borchers, J. A.; Grutter, A. J., Effects of field annealing on MnN/CoFeB exchange bias systems. *Phys. Rev. Mater.* **2019,** *3* (6), 064413.
2. Kirby, B. J.; Kienzle, P. A.; Maranville, B. B.; Berk, N. F.; Krycka, J.; Heinrich, F.; Majkrzak, C. F., Phase-sensitive specular neutron reflectometry for imaging the nanometer scale composition depth profile of thin-film materials. *Curr. Opin. Colloid In.* **2012,** *17* (1), 44-53.
3. Maranville, B.; Ratcliff II, W.; Kienzle, P., reductus: a stateless Python data reduction service with a browser front end. *J. Appl. Crystallogr.* **2018,** *51* (5), 1500-1506.
